# Effects of Vitamin D Supplementation on Omentin-1 and Spexin Levels, Inflammatory Parameters, Lipid Profile, and Anthropometric Indices in Obese and Overweight Adults with Vitamin D Deficiency under Low-Calorie Diet: A Randomized Placebo Controlled Trial

**DOI:** 10.1155/2020/3826237

**Published:** 2020-11-10

**Authors:** Elhameh Cheshmazar, Agha Fatemeh Hosseini, Bahareh Yazdani, Elham Razmpoosh, Mitra Zarrati

**Affiliations:** ^1^Department of Nutrition, School of Public Health, Iran University of Medical Sciences, Tehran, Iran; ^2^Department of Biostatistics, School of Public Health, Iran University of Medical Sciences, Tehran, Iran; ^3^Neonatal Screening Department of Nilou Laboratory, Tehran, Iran; ^4^Nutrition and Endocrine Research Center, Research Institute for Endocrine Sciences, Shahid Beheshti University of Medical Sciences, Tehran, Iran; ^5^Integrative Oncology and Quality of Life Department, Breast Cancer Research Center, Motamed Cancer Institute, ACECR, Tehran, Iran

## Abstract

**Background and Aims:**

Improved vitamin D levels can have a favorable effect on some metabolic variables. The objective of the current study was to determine the effects of vitamin D supplementation during a weight-loss intervention on the levels of omentin-1, spexin, lipid profiles, and inflammatory factors in obese and overweight participants.

**Methods and Materials:**

In this double-blind placebo-controlled randomized clinical trial, 70 overweight and obese participants with vitamin D deficiency (25(OH)D ≤ 20 nmol/L) were assigned into the intervention (a daily dose of 2,000 IU vitamin D + low-calorie diet) and placebo (placebo + low-calorie diet) groups for 8 weeks. Anthropometric parameters, serum levels of 25-hydroxy vitamin D (25(OH)D), lipid profiles, omentin-1 and spexin levels, high-sensitivity C-reactive protein (hs-CRP), and soluble intercellular adhesion molecule-1 (sICAM-1) concentrations were assessed before and after the intervention.

**Results:**

Vitamin D supplementation after the intervention led to a significant decrease in triglycerides (TG) (*P* = 0.02), very-low-density lipoprotein-cholesterol (VLDL-C) (*P* = 0.02), and hs-CRP (*P* = 0.03) concentrations and a significant increase in the serum vitamin D level (*P* < 0.001). Furthermore, after adjusting for baseline values, age, and baseline BMI, the levels of serum high-density lipoprotein-cholesterol (HDL-C) (*P* = 0.01) increased significantly, and a significant reduction was observed in the concentration of sICAM-1 (*P* = 0.01) in the intervention group. However, we did not find any significant difference in serum omentin-1 and spexin concentrations between the groups after intervention.

**Conclusions:**

Vitamin D supplementation along with a low-calorie diet (LCD) program for 8 weeks significantly decreased the inflammatory markers in obese individuals, while it did not alter serum omentin-1 and spexin concentrations.

## 1. Introduction

Obesity is now a considerable public health problem, which is mainly caused by genetic factors, increased calorie intake, and the lack of physical activity [1]. Obesity and overweight have been proved to be associated with several comorbidities including diabetes mellitus, cardiovascular diseases (CVD), and different types of cancers such as breast and colon cancer, which are all caused by the inflammatory responses in the body [2]. Obesity and overeating are associated with accumulation and expansion of adipose tissue, so hypertrophic adipocytes may play an important role in the onset of inflammation by disrupting the balance of inflammatory and anti-inflammatory factors [3]. In fact, recent evidence has shown that vitamin D can modulate the secretions of many adipokines [4], which are mainly secreted by adipose tissue (AT). These adipokines mainly include spexin and omentin-1 which play an important role in several biological processes, such as regulating the uptake of long-chain free fatty acids and the body fat storage, modulating food intake and energy metabolism [5], as well as regulating the anti-inflammatory, antidiabetic, antiatherogenic, and insulin-sensitizing effects in the body [6]. Therefore, there are many disorders known to be related to increased AT mass and the subsequent incidence of inflammation [7]. Different effects of vitamin D supplementation may be observed on adipokine homeostasis [8]. The presence of hypovitaminosis may impair the regulation of antioxidant and anti-inflammatory pathways [9]. Besides, it has been observed that a low-calorie diet could alleviate the inflammatory status and modulate the adipokine levels. Regulation of inflammatory and anti-inflammatory markers balance and increase insulin sensitivity after weight loss has been associated with a decrease in the prevalence of chronic diseases [10]. Improvements in adipokine profile with weight loss of 5–10% have been observed in previous studies [11–13]. There are previous animal and human studies on obesity and metabolic syndrome (MetS) that have reported various effects of vitamin D supplementation on some adipokines and the biomarkers of inflammation, blood pressure, glycemic control, and lipid profiles in obesity [9, 14].

To our knowledge, this is the first randomized double-blind placebo-controlled trial of vitamin D supplementation on omentin-1 and spexin levels in obese and overweight adults during low-calorie diet with vitamin D deficiency.

Given the modulatory and anti-inflammatory effects of vitamin D, we hypothesized that the low-calorie diet programs along with vitamin D supplementation could improve serum omentin-1 and spexin levels and modulate the inflammatory markers, lipid profiles, and anthropometric parameters in overweight and obese individuals. The purpose of this randomized placebo-controlled trial was to investigate the effects of a 2,000 IU of vitamin D_3_ supplementation per day for 8 weeks on some serum adipokines including omentin-1 and spexin levels, inflammatory markers, lipid profile, and anthropometric parameters along with a low-calorie diet program in overweight and obese participants who had vitamin D deficiency.

## 2. Materials and Methods

### 2.1. Study Design and Participants

**Figure 1:**
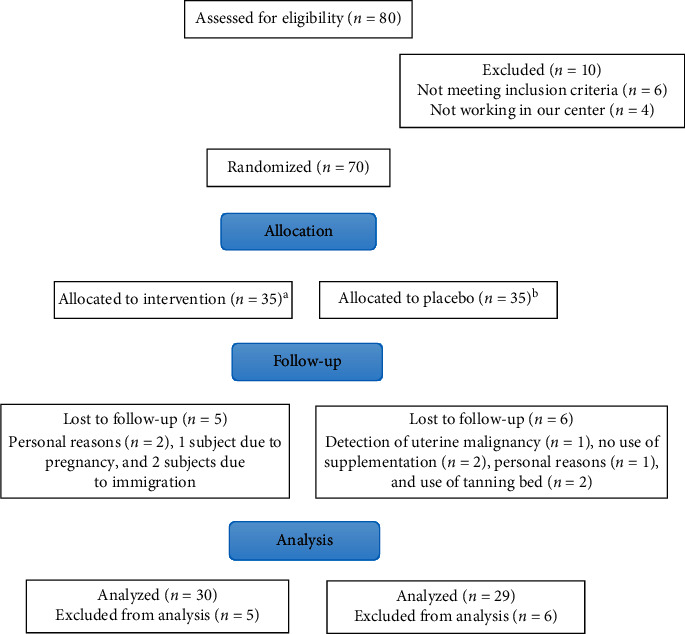
Summary of the patient flow diagram.

This randomized double-blind placebo-controlled clinical trial was conducted at the Nutrition Clinic of Iran University of Medical Sciences (IUMS). Seventy obese and overweight participants (25 men and 45 women) aged 20–50 years and with a baseline body mass index (BMI) of 25–35 kg/m^2^ were recruited for the study. Inclusion criteria were having a serum 25(OH)D level of less than 20 ng/mL, not taking any glucose and lipid-lowering drugs, and willing to participate in the investigation. The exclusion criteria were having any metabolic diseases, diabetes or CVD, autoimmune diseases, any types of cancers, renal or hepatic and infection diseases, women with polycystic ovary syndrome, smoking, the alcohol consumption, taking immune system suppressor drugs in 6 months prior to the study initiation as well as having a weight loss diet program in 3 months prior to the research, taking any multimineral supplements or vitamin D supplements, the use of tanning bed during the study or in 3 months before the study initiation, and having no interests for participating in the research. The overall diagram of the study research is shown in Figure 1. At the onset of the study, all the participants were randomized into two groups of vitamin D intervention and placebo. Individuals were matched one by one according to their gender and BMI ranges. Randomization assignment was conducted using computer-generated random numbers as blindness by trained personnel. A balanced-blocked randomization list stratified by gender and BMI (block 1: BMI 25–29.9 kg/m^2^ female, block 2: BMI: 30–35 kg/m^2^ female, block 3: BMI 25–29.9 kg/m^2^ male, and block 4: BMI 30–35 kg/m^2^ male). Participants in the vitamin D group received an oral 2,000 IU of vitamin D supplement (Schiff & Company, USA) per day for 8 weeks and the placebo group received capsules of edible paraffin oil for 8 weeks which were similar in their shapes and sizes to vitamin D supplements. To prevent the effects of seasonal variations on vitamin D levels, this study was conducted in the autumn and winter.

Both groups received a weight loss program at the baseline of the study. The related diet consisted of 12–15% calories of protein, 30–35% of fat, and 55–60% of carbohydrates which was estimated individually based on each participant's current age, weight, height, and gender. We asked every participant to come to the clinic every two weeks for weight monitoring and receiving their supplements for the upcoming two weeks. To increase compliance, all the participants received short messages on their cell phones every week to remind them of the adherence to their weight loss diet program and taking the supplements. To assess their dietary intakes, every participant completed a 3-day dietary record: at baseline, week 3, and at the end of the trial. Modified Nutritionist-4 software program (First Databank, San Bruno, CA) was used to determine the macro- and micronutrient intakes. Physical activity was described as metabolic equivalents in hours per day and assessed three times by Long International Physical Activity (IPAQ) questionnaire during the study. Individuals were requested to have a 30-minute walk per day and not to consume any supplements other than the one provided to them by the investigators.

### 2.2. Sample Size Calculation

The mean ± SD of omentin-1 and sICAM-1 were estimated from previous studies [15, 16]. By assuming a type one error (a) of 0.05 and type two error (b) of 0.20 (power = 80%), after considering a 10% dropout rate, a sample size of 35 participants per group was finally calculated.

### 2.3. Measurements

The primary outcomes were followed as adipokines including omentin-1 and spexin and inflammatory factors including hs-CRP and sICAM-1. Secondary outcomes were lipid profile consisting of LDL-C, HDL-C, TG, and TC, as well as VLDLD-C and anthropometric indices including BMI, weight, fat, and muscle percent, waist circumference (WC), and visceral fat (VF).

#### 2.3.1. Assessment of Anthropometric Measures

Weight (nearest to 0.1 kg) and height (nearest to 0.1 kg) with light clothes were estimated. Fat percent and muscle percent were measured using a standard scale (Omron-Body Composition Monitor-BF511, Kyoto, Japan) and Seca Messband 206 in a fasting status at baseline and after the 8 weeks. Furthermore, BMI was calculated as weight in kg divided by the square of height in meter. WC and VF were estimated by ViScan device (Abdominal Fat Analyser AB-140, Tanita). The blood pressure of participants was measured by a digital blood pressure device (Omron, HEM-FL31) and we were confident that every individual was relaxed at least for 10–15 minutes before the measurement.

#### 2.3.2. Sun Exposure Questionnaire

The participants' exposure to sunlight was assessed before and after the intervention through a validated questionnaire. In the questionnaire about the duration of sunlight exposure (the average minutes (min)/hours (h) of a usual day in the previous week), use of sunscreen, the types of coating, and the noncovered part of the body during sun exposure were asked [17]. The duration of sun exposure was classified as 0–10 min; 10 min–1 h; 1-2 h; and >2 h. Moreover, the time of exposure to sunlight during a day was also asked and classified as follows: 7 am–10 am; 10 am–3 pm; and 3 pm–5 pm. Because of the intense sunlight, the highest score was the exposure of sunlight at 10–3 o'clock [18].

#### 2.3.3. Biochemical Assessment

Blood samples (10 mL) were taken from every participant after overnight fasting at baseline and at the end of 8 weeks at the nutrition clinic of IUMS. 25(OH)D concentration was evaluated using a commercial ELISA kit (Immunodiagnostic Systems). The inter- and intra-assay coefficients of variability (%CV) for serum 25(OH)D tests ranged from 5% to 7.5%. Serum spexin and omentin-1 (MyBioSource, San Diego, USA), sICAM-1 (Eagle Biosciences, USA) levels were quantified using enzyme-linked immunosorbent assay (ELISA) kits with inter- and intra-assay %CV of 8.5 and 9.6%, respectively. To determine the levels of fasting blood sugar (FBS), serum TG, TC, LDL-C and HDL-C, VLDL-C, and hs-CRP, we used related enzymatic kits (DIALAB Kit, Fara Samed Company, Iran).

### 2.4. Statistical Analysis

Data were statistically analyzed using the Statistical Package for Social Sciences (SPSS, version 22, Chicago, IL, USA). Using the one-sample Kolmogorov–Smirnov test, it was found that all primary and secondary data had normal distribution. Independent sample *t*-test was used for the comparison of continuous variables, dietary intakes, and anthropometric measures and Pearson's chi-square test for comparing the categorical variables between the 2 groups. We applied paired-sample *t*-test to establish within-group differences (pre- and posttreatment). Independent *t*-test was used to evaluate the effect of vitamin D supplementation on adipokines, inflammatory markers, and lipid concentrations in the intervention and placebo groups. The results were considered significant if the *P* value was <0.05. To assess the effects of some confounders, we adjusted all analyses using ANCOVA test.

### 2.5. Ethical Approval

The study protocol was approved by the ethics committee of IUMS and registered at the Iranian Registry of Clinical Trial. This investigation was conducted according to the principles of the Declaration of Helsinki and carefully explained to all participants before obtaining the informed consent form.

## 3. Results

Of the 80 individuals screened for eligibility, 70 participants met the inclusion criteria and were allocated equally into 2 groups of 35. However, 84.2% of all the participants including 29 individuals in the placebo and 30 individuals in the vitamin D group completed the study (Figure 1). In fact, 5 participants in the vitamin D group were lost to follow-up due to personal reasons (*n* = 2), pregnancy (*n* = 1), and immigration (*n* = 2). In addition, the other 6 participants who were lost to follow-up in the study were in the placebo group, and it was due to uterine malignancy (*n* = 1), personal reasons (*n* = 1), use of tanning bed (*n* = 2), and the lack of sufficient vitamin D supplement consumption (*n* = 2). No adverse effects of interventions were reported. General characteristics are shown in Table 1. The mean age and height of all patients were estimated to be 37.57 ± 8.5 years and 159.8 ± 31.11 cm, respectively.

### 3.1. Body Composition

In both groups, anthropometric indices (height, body weight, BMI, fat percent, and WC) except muscle percent and VF significantly decreased after 8 weeks of intervention, but changes between vitamin D and placebo groups were not significant. Systolic blood pressure (SBP) and diastolic blood pressure (DBP) were not significant within and between the two groups before and after the treatment.  Table 2 summarizes the selected data of participants at the beginning and at the end of the study.

### 3.2. Food Intake

According to the 3-day dietary records, no statistically significant differences were observed between the two groups in terms of macro- and micronutrients (Table 3).

At the end of the study, it was found that dietary intakes of vitamin D (independent of vitamin D supplementation) were slightly diminished in both groups without any significant differences between the groups (Table 3).

### 3.3. Main Variable

After the 8 weeks of vitamin D supplementation, serum 25(OH)D concentrations increased in the vitamin D group (vitamin D vs. placebo groups: 16.8 ± 8.8 vs. 0.6 ± 1.4 ng/mL, *P* < 0.001). Vitamin D supplementation resulted in a significant reduction in serum triglycerides (vitamin D vs. placebo groups: −37.3 ± 75.7 vs. 2.2 ± 64.4 mg/dL, *P* = 0.02), VLDL-C levels (vitamin D vs. placebo groups: −16.9 ± 34.4 vs. 1 ± 29.2 mg/dL, *P* = 0.02), and hs-CRP concentration (vitamin D vs. placebo groups: −850 ± 0.321 vs. −106.54 ± 5058.3 ng/mL, *P* = 0.03) (Table 4). When the analyses were adjusted for baseline levels of variable, age, and baseline BMI, a significant increase was observed in HDL-C concentration (8.3 ± 1.6 vs. 0.52 ± 1.6 mg/dL, *P* = 0.01), and *P* value of triglycerides (*P* = 0.01), VLDL-C (*P* = 0.01), and hs-CRP (*P* = 0.01) was decreased (Table 5). We did not observe a significant effect of vitamin D on serum omentin-1, spexin, sICAM-1, FBS, total-cholesterol, and LDL-C concentrations (Table 4). When the analyses were adjusted for baseline levels, age, and baseline BMI, an only significant decrease was observed in sICAM-1 levels (−316.8 ± 76.3 vs. −209.3 ± 77.6 ng/dL, *P* = 0.01) (Table 5).

## 4. Discussion

The current study indicated that vitamin D supplementation for 8 weeks in obese and overweight participants with vitamin D deficiency who had a low-calorie diet program led to a significant reduction in serum levels of hs-CRP, sICAM-1, triglycerides, and VLDL-cholesterol. However, we did not find any considerable effect of vitamin D supplementation on serum omentin-1 and spexin concentrations or anthropometric indices. To the best of our knowledge, this is the first controlled trial study that assessed the effect of vitamin D supplementation on omentin-1 and spexin levels in obese and overweight participants with vitamin D deficiency undergoing a low-calorie diet.

Omentin-1 is an adipocytokine secreted from adipose tissue and may be inversely correlated with obesity and insulin resistance or impaired glucose tolerance [15, 19, 20]. Our findings show that omentin-1 and spexin levels remained unchanged in both intervention and placebo groups. Jafari et al. [19] showed that daily consumption of 2,000 IU vitamin D-fortified yogurt for 12 weeks in postmenopausal women with type 2 diabetes improved omentin-1 level. Anti-inflammatory, antiatherogenic, and antidiabetic effects can be attributed to omentin-1 [19]. In another study, a linear relationship was observed between omentin-1 level and vitamin D in postmenopausal women with or without osteoporosis. This association was stronger in subjects with normal vitamin D compared with insufficient levels [21]. Moreno-Navarrete et al. [22] indicated that the inverse relationship between obesity and omentin-1 may be due to a downregulation of insulin gene expression. Although the desirable effects of omentin-1 have been observed in only a few studies, further studies are needed to confirm these effects and to identify more omentin-1.

In the current study, no significant differences were observed regarding the spexin levels between groups, at the end of the study. Investigations showed that spexin concentrations were found to be significantly lower in obese children [23] and adults with severe obesity [5]. Furthermore, a downregulation of spexin gene expression was identified in obese human fat tissue compared with nonobese individuals [24]. Decreased expression of spexin gene due to elevated serum leptin levels in obese individuals is associated with impaired intake foods and energy metabolism [5]. On the other hand, it has been suggested that spexin can act as a satiety factor [5]. Thus, adipose tissue accumulation and reduction of spexin in obesity may impair control of food intake and lack of awareness of satiety [5]. There was no significant difference between the two groups in terms of weight and fat percent; it might be an interpretation for no significant change in spexin levels after the intervention.

Vitamin D supplementation in obese and overweight adults with vitamin D deficiency under a low-calorie diet reduced hs-CRP concentrations significantly. Our findings are consistent with those of Asemi et al. [25] who reported that vitamin D supplementation (400 IU/d) resulted in a significant decrease in serum hs-CRP in 48 pregnant women aged 18–40 y old at 25 weeks of gestation. In addition, in Sharifi et al.'s study [26], supplementation with one oral pearl consisting of 50,000 IU vitamin D3, every 14 for 120 days, had beneficial effects on hs-CRP level.

Contrary to our finding, a previous study showed that oral cholecalciferol supplementation (100,000 IU bolus followed by 4,000 IU daily) for 16 weeks among healthy overweight and obese individuals with vitamin D deficiency had no significant effects on proinflammatory markers and the nuclear factor kappa B (NF-*κ*B) activity. They also mentioned that despite the significant increase found in serum 25(OH)D concentrations in the vitamin D group, no considerable alterations were observed in hs-CRP levels before and after the adjustment [27]. Inconsistent with our finding with respect to the significant changes in hs-CRP levels, the results of a recent meta-analysis have shown that vitamin D supplementation had no significant influence on hs-CRP levels in overweight and obese population [28]. In general, principal physiologic properties of calcitriol on cardiomyocytes, vascular smooth muscle cells, and the vascular endothelium might be related to the protective effects of vitamin D on cardiovascular disease [29]. In fact, it has been shown that 1,25-dihydroxy vitamin D, as the active form of vitamin D, could reduce the hs-CRP concentrations via inhibiting the NF-*κ*B pathway [30]. We observed that before the adjusted analysis, the supplementation of vitamin D had no significant effect on sICAM-1. However, a significant decrease was observed for sICAM-1 after adjusting for baseline levels, BMI, and age as the confounding variables. With regard to the relation between vitamin D and soluble endothelial cell adhesion molecules as the biomarkers of endothelial dysfunction, it was found that sICAM-1 might be associated with hypovitaminosis D [31]. In line with our study finding, vitamin D supplementation (50,000 IU vitamin D weekly for 12 weeks and then with the same dose every 3 weeks for 3 months) in end-stage renal disease (ESRD) patients, reduced the serum levels of ICAM and VCAM in the treatment group significantly [31]. Another study indicated that intramuscular administration 300000 IU vitamin D supplement in type 2 diabetes (T2D) patients with ischemic heart disease had no significant changes in the levels of ICAM-1 and VCAM-1 [32]. Contrary to our results, a previous parallel-group, double-blind, placebo-controlled randomized controlled trial showed that a 12-month intervention of vitamin D supplementation (400 IU or 1,000 IU per day) had no effect on inflammatory markers such as hs-CRP and sICAM-1 in healthy postmenopausal women [33]. Elevated levels of ICAM reflect the increased risk of CVD. In fact, the existence of cardiovascular risk factors such as insulin resistance, atherogenic dyslipidemia, hypertension, and inflammation in obese population is accompanied by the elevated levels of soluble adhesion molecules [34]. In view of the fact, normal vitamin D levels and a decrease in s-ICAM-1 concentration can be effective in improving endothelial function and reducing the risk of CVD [31].

In the present study, anthropometric indices including height, body weight, BMI, fat percent, and WC significantly decreased after 8-week intervention in both groups, but none of the variable indicated significant differences between vitamin D and placebo groups. Consistent with our study, Jafari-Sfidvajani et al. [35] did not find significant differences in anthropometric indices among women with polycystic ovary syndrome (PCOS) patients in two groups including (50,000 IU/week) vitamin D_3_ + weight-loss diet or placebo + weight-loss diet. In addition, in line with our results, Lorvand Amiri et al. [36] reported no significant differences in anthropometric parameters among 73 nonalcoholic fatty liver disease participants with insufficient concentrations of 25(OH)D at baseline who received 25 *μ*g vitamin D_3_/day or placebo for 12 weeks while participating in a weight-loss program. In agreement with our study, Salehpour et al. [37] accomplished a 12-week intervention and stated that cholecalciferol supplementation did not have any significant effect on body composition in both healthy and obese women compared to the placebo group, but a low significant decrease was observed in body fat mass. However, Wamberg et al. [38] who evaluated the long-term effects of vitamin D supplementation on body fat accumulation, inflammatory markers, and metabolic risk factors in obese adults with low vitamin D levels for 26 weeks did not observe any significant changes on body weight and body composition. Karonova et al. [39] reported that serum 25(OH)D level was inversely correlated with body weight, WC, and BMI in females but not in men. Furthermore, similar trends were found in the analyses of a cohort study that reported an inverse association between serum vitamin D and weight, BMI, and fat mass [40]. We assume that the results of the absence of significant findings regarding vitamin D supplementation and anthropometric indices could be related to the low-calorie diet program that was given to all the participants.

Findings from the current study revealed that the administration of vitamin D supplements among vitamin D-deficient obese and overweight participants led to a significant decrease in serum triglycerides and VLDL-cholesterol levels and a significant increase in serum HDL-C concentration. The correlation between hypovitaminosis D and the increased risk of major adverse cardiovascular disease (CVD) events has been strongly proven in epidemiological studies [41, 42]. In line with our study, Lorvand Amiri et al. [36] found that vitamin D supplementation in patients with nonalcoholic fatty liver disease significantly reduced triglycerides levels and increased HDL-cholesterol concentrations in the intervention group. In compliance with our findings, Major et al. [43] revealed that a 15-week weight-reducing program along with a calcium plus vitamin D supplementation resulted in beneficial effects on lipoprotein profiles among overweight and obese women. The results of Nagpal et al. [44] study showed that lipid profiles of middle-aged, centrally obese, and healthy participants remained unchanged after a 6-week supplementation of cholecalciferol. Notably, the results of a previous meta-analysis indicated that vitamin D supplementation led to a significant decrease in LDL-cholesterol concentrations, while no considerable improvements were observed regarding the levels of total cholesterol, HDL-cholesterol, and triglycerides [45]. The heterogeneity between results among different studies may be related to the difference in dose and duration of vitamin D supplementation, various sample sizes, different characteristics of study populations as well as various measuring techniques and different types of formulation used in preparing the supplements, and different baseline vitamin D levels of participants.

The strength of the present study is that this is the first randomized placebo-controlled trial to investigate the effect of vitamin D supplementation on omentin-1 and spexin concentration in obese and overweight individuals with vitamin D deficiency; moreover, vitamin D levels improved in the intervention group compared to the control group. The limitations of the present study should be mentioned; the small sample size and the short duration of vitamin D intervention are the main limitations; in addition, all participants received a weight-loss intervention, so we were unable to determine the effects of vitamin D_3_ supplementation and low-calorie diet program separately. On the contrary, measuring the gene expression of omentin-1 and spexin could be useful in analyzing the results, but it was not possible due to time constraints. Thus, additional randomized clinical trial studies with larger populations, longer duration on intervention, and measuring the gene expression of adipokines are required to determine the exact association of vitamin D intervention and low-calorie diet with body composition, adipokines, lipid profiles, and inflammatory markers in obese and overweight participants.

## 5. Conclusion

This study indicated a significant decrease in hs-CRP, sICAM-1, TG, and VLDL-C and a significant increase in HDL-C and serum 25(OH)D levels by vitamin D supplements in the intervention group, but no significant alterations were detected on omentin-1 and spexin concentrations; hence, further studies are needed to show vitamin D effects on these adipokines and confirm results.

## Figures and Tables

**Table 1:** General characteristics of study participants.

	Placebo group^1^ (*n* = 29)	Vitamin D group^2^ (*n* = 30)	*P* ^*∗*^
Age (y)^a,e^	36.82 ± 7.65	38.30 ± 9.34	0.5
Gender^d^			0.3
Female^b^	21 (72.4)	18 (60)	
Male^b^	8 (27.6)	12 (40)	
Height (m)^a,e^	154.5 ± 143.13	165.03 ± 9.17	0.1
Sun exposure (score)^f^			
Baseline^c^	4.4 (1.8, 6.9)	4.9 (2.1, 7)	0.7
End of the trial^c^	3.9(1, 7)	4.4(1.7, 5.8)	0.8
Physical activity (IPAQ-METS score)^g^			
Baseline^c^	630(443,1299)	682.5(470.25,1903.5)	0.8
End of the trial^c^	600(393.5,1400)	827.5(415.7,1925)	0.5

^1^Received placebo per day during 8 weeks. ^2^Received 2,000 IU vitamin D per day during 8 weeks. ^*∗*^Significant difference (*P* < 0.05). ^a^Data are presented as means ± SDs and frequency (%)^b^ or median (interquartile range)^c^. ^d^Obtained from chi-square. ^e^Obtained from independent sample Student's *t*-test. ^f^Obtained from Fisher's exact test. ^g^Obtained from Mann–Whitney *U* test.

**Table 2:** Comparison of the anthropometric index changes within and between groups after the intervention.

	Placebo group^1^ (*n* = 25)				Vitamin D group^2^ (*n* = 30)				
	Baseline	End of the trial	Change	*P* ^*a*^	Baseline	End of the trial	Change	*P* ^*a*^	*P* ^*b*^
Weight (kg)	88.05 ± 13.32	84.10 ± 19.94	−3.95 ± 11	<0.001	86.33 ± 14	85.11 ± 13.06	−1.22 ± 2.2	<0.001	0.58
BMI (kg/m^2^)	32.16 ± 4.35	31.05 ± 4.48	−1.11 ± 0.95	<0.001	32.47 ± 4.05	31.06 ± 4.06	−1.41 ± 0.97	<0.001	0.7
Fat percent	39.77 ± 9.18	38.80 ± 9.52	−0.96 ± 1.81	0.04	40.91 ± 8	39.41 ± 8.44	−1.49 ± 1.97	0.03	0.7
Muscle percent	26.02 ± 5.85	26.56 ± 5.97	0.53 ± 1.30	0.3	26.87 ± 5.40	27.40 ± 5.56	0.73 ± 1.7	0.2	0.35
Waist circumference (cm)	115.89 ± 12	111.72 ± 11.9	−4.17 ± 1.61	<0.001	116 ± 10.56	112.3 ± 11.06	−3.66 ± 1.19	<0.001	0.52
Visceral fat	10.36 ± 3.20	10.08 ± 3.06	−0.27 ± 0.77	0.07	10.27 ± 3.37	10.23 ± 3.35	−0.48 ± 0.71	0.06	0.65

BMI: body mass index. ^1^Received placebo per day during 8 weeks. ^2^Received 2,000 IU vitamin D per day during 8 weeks. Significant difference (*P* < 0.05). Data are presented as means ± SDs. *P*^*a*^: comparison of within-group changes (paired-sample *t*-test). *P*^*b*^: comparison of between-group changes (independent *t*-test).

**Table 3:** Dietary intakes of study participants throughout the study.

	Placebo group^1^ (*n* = 29)	Vitamin D group^2^ (*n* = 30)	*P* ^*∗*^
Calorie at study baseline (kcal/d)	3302.7 ± 889.2	3373.2 ± 1501	0.8
Calorie at the end of the trial (kcal/d)	2630.4 ± 964.5	2444.3 ± 1079.6	0.4
Carbohydrate at study baseline (gr/d)	282.2 ± 74.6	299.9 ± 121.7	0.6
Carbohydrate at the end of the trial (gr/d)	235 ± 83.4	243.8 ± 111.5	0.7
Protein at study baseline (gr/d)	89.2 ± 37	84.2 ± 38.1	0.5
Protein at the end of the trial (gr/d)	82 ± 42.3	69.4 ± 37.5	0.6
Fat at study baseline (gr/d)	198.4 ± 93.1	211.3 ± 127.3	0.8
Fat at the end of the trial (gr/d)	143.5 ± 89.7	136.3 ± 78.2	0.8
SAFs at study baseline (gr/d)	36.2 ± 15.2	38 ± 21.1	0.7
SAFs at the end of the trial (gr/d)	29.2 ± 12.6	28 ± 14	0.7
PUFA at study baseline (gr/d)	114.1 ± 59.7	107.2 ± 67.8	0.3
PUFA at the end of the trial (gr/d)	75.7 ± 56.2	70.2 ± 55.5	0.5
MUFA at study baseline (gr/d)	47.7 ± 21.7	63.3 ± 57.5	0.8
MUFA at the end of the trial (gr/d)	39.5 ± 24.8	39.3 ± 28.1	0.6
Vitamin D at study baseline (*μ*g/d)	0.44 ± 1	1.14 ± 1.8	0.5
Vitamin D at the end of the trial (*μ*g/d)	0.33 ± 0.96	1.09 ± 2.3	0.4
Calcium at study baseline (mg/d)	644 ± 411.5	600 ± 376.5	0.6
Calcium at the end of the trial (mg/d)	474.8 ± 343.3	523.8 ± 332.5	0.4
Iron at study baseline (mg/d)	17.5 ± 6	15.37 ± 7	0.1
Iron at the end of the trial (mg/d)	14.4 ± 6.3	12.15 ± 5	0.1
Mg at study baseline (mg/d)	237.7 ± 134.8	22 ± 128.9	0.4
Mg at the end of the trial (mg/d)	195.9 ± 100.2	166 ± 83.9	0.2
Na at study baseline (mg/d)	1721.2 ± 2248.9	1344.9 ± 1341.2	0.8
Na at the end of the trial (mg/d)	1265.3 ± 2003.7	921.6 ± 740.5	0.9
Dietary fiber at study baseline (gr/d)	21.9 ± 16.3	17.7 ± 10.8	0.4
Dietary fiber at the end of the trial (gr/d)	19.1 ± 16.8	14.4 ± 9.4	0.5

SFAs: saturated fatty acids; PUFAs: polyunsaturated fatty acids; MUFAs: monounsaturated fatty acids; Na: sodium; Mg: magnesium. ^1^Received placebo per day during 8 weeks. ^2^Received 2,000 IU vitamin D per day during 8 weeks. ^*∗*^Significant difference (*P* < 0.05). Data are presented as means ± SDs. ^*∗*^Obtained from independent-sample *t*-test.

**Table 4:** Means (±standard deviations) of glycemic control and markers of cardiometabolic risk at baseline and after the 8-week intervention in patients with overweight and obese that received vitamin D supplement or placebo.

	Placebo group^1^ (*n* = 29)				Vitamin D group^2^ (*n* = 30)				
	Baseline	End of the trial	Change	*P* ^*a*^	Baseline	End of the trial	Change	*P* ^*a*^	*P* ^*b*^
Serum 25(OH)D (ng/mL)	19.3 ± 3.6	19.9 ± 3.5	0.6 ± 1.4	0.6	19.8 ± 2.6	36.6 ± 9.8	16.8 ± 8.8	0.02	<0.001
Omentin-1 (mg/dL)	201.7 ± 197.6	216.9 ± 179.4	15.2 ± 229.1	0.6	255 ± 267.1	273.5 ± 280.11	18.49 ± 280.6	0.5	0.75
Spexin (pg/mL)	8169.4 ± 6753.2	8355.1 ± 6109	185.7 ± 6045.7	0.9	8533.3 ± 6198.2	8738.2 ± 5959.3	204.9 ± 5325.8	0.6	0.8
hs-CRP (ng/mL)	4014.54 ± 4730.89	3908 ± 5100.4	−106.54 ± 5058.3	0.6	3606 ± 0.522	2756 ± 0.344	−850 ± 0.321	0.02	0.03
sICAM-1(ng/L)	835.8 ± 687.1	644.4 ± 368.5	−191.3 ± 729.2	0.4	837.8 ± 578.7	530.5 ± 389.3	−307.2 ± 466.6	0.01	0.07
FBS (mg/dL)	101 .75 ± 10.84	104.20 ± 25.12	2.4 ± 19.7	0.09	100.70 ± 9.86	96.96 ± 10.84	−3.7 ± 10.71	0.07	0.2
Triglycerides (mg/dL)	180.62 ± 74.13	182.82 ± 87.76	2.2 ± 64.4	0.7	172.36 ± 94.33	135.06 ± 64.5	−37.3 ± 75.7	0.01	0.02
HDL-C (mg/dL)	51.89 ± 10.33	52.31 ± 10.51	0.41 ± 8.8	0.8	46.80 ± 13.46	52.2 ± 14.31	5.4 ± 10.58	<0.001	0.08
LDL-C (mg/dL)	137.3 ± 44.48	141.5 ± 38.34	4.24 ± 37.38	0.59	139.50 ± 74.60	130.20 ± 54.48	−9.3 ± 39.24	0.45	0.08
Total cholesterol (mg/dL)	212.34 ± 34.11	206.5 ± 37.31	−5.8 ± 41.34	0.44	205.13 ± 77.93	190.03 ± 73.21	−15.10 ± 24.90	0.06	0.6
VLDL-C (mg/dL)	82.1 ± 33.6	83.1 ± 39.8	1 ± 29.2	0.7	78.3 ± 42.8	61.3 ± 29.3	−16.9 ± 34.4	0.01	0.02
SBP (mmHg)	11.51 ± 1.47	11.06 ± 1.74	−0.44 ± 1.53	0.12	11.26 ± 1.72	11.33 ± 1.65	0.06 ± 1.16	0.75	0.55
DBP (mmHg)	8.03 ± 1.68	7.84 ± 1.32	−0.18 ± 1.12	0.58	8.30 ± 1.55	8.22 ± 1.44	−0.8 ± 1.25	0.76	0.8

FBS: fasting blood sugar; HDL-C: high-density lipoprotein-cholesterol; LDL-C: low-density lipoprotein-cholesterol; VLDL-C: very-low-density lipoprotein-cholesterol; SBP: systolic blood pressure; DBP: diastolic blood pressure. ^1^Received placebo per day during 8 weeks. ^2^Received 2,000 IU vitamin D per day during 8 weeks. ^*∗*^Significant difference (*P* < 0.05). *P*^*a*^: comparison of within-group changes (paired-sample *t*-test). *P*^*b*^: comparison of between-group changes (independent *t*-test). Data are presented as means ± SDs.

**Table 5:** Adjusted changes in metabolic variables in patients with overweight and obese that received either vitamin D supplement or placebo.

	Placebo group^1^ (*n* = 29)	Vitamin D group^2^ (*n* = 30)	*P* ^*∗*^
Serum 25(OH)D (ng/mL)	0.2 ± 0.87	15 ± 0.6	<0.001
Omentin-1 (ng/L)	12.4 ± 0.11	16.5 ± 0.11	0.1
Spexin (pg/mL)	122.3 ± 0.15	184 ± 0.14	0.3
hs-CRP (ng/mL)	−95.13 ± 754.3	−783.96 ± 832.12	0.01
sICAM-1(ng/L)	−209.3 ± 77.6	−316.8 ± 76.3	0.01
FBS (mg/dL)	103.2 ± 2.8	97.8 ± 2.8	0.1
Triglycerides (mg/dL)	5.7 ± 11.9	38.9 ± 11	0.01
HDL-cholesterol (mg/dL)	0.52 ± 1.6	8.3 ± 1.6	0.01
LDL-cholesterol (mg/dL)	4.9 ± 0.4	−8.4 ± 0.4	0.1
Total cholesterol (mg/dL)	−7.4 ± 16.8	−18.5 ± 0.5	0.1
VLDL-C (mg/dL)	2.1 ± 41.2	15.3 ± 0.9	0.01

FBS: fasting blood sugar; HDL-C: high-density lipoprotein-cholesterol; LDL-C: low-density lipoprotein-cholesterol; and VLDL-C: very-low-density lipoprotein-cholesterol. ^1^Received placebo per day during 8 weeks. ^2^Received 2,000 IU vitamin D per day during 8 weeks. ^*∗*^Significant difference (*P* < 0.05). ^*∗*^Obtained from ANCOVA test adjusted for baseline values, age, and baseline BMI. Data are presented as means ± SDs.

## Data Availability

The datasets used during the current study are available from the corresponding author upon reasonable request.
